# Pancreatic cancer chemo-resistance is driven by tumor phenotype rather than tumor genotype

**DOI:** 10.1016/j.heliyon.2018.e01055

**Published:** 2018-12-17

**Authors:** Mirna Swayden, Juan Iovanna, Philippe Soubeyran

**Affiliations:** Centre de Recherche en Cancérologie de Marseille (CRCM), INSERM U1068, CNRS UMR 7258, Aix-Marseille Université and Institut Paoli-Calmettes, Parc Scientifique et Technologique de Luminy, Marseille, France

**Keywords:** Cancer research, Oncology, Cell biology, Molecular biology

## Abstract

Pancreatic Ductal Adenocarcinoma (PDAC) is one of the deadliest forms of cancer. A major reason for this situation is the fact that these tumors are already resistant or become rapidly resistant to all conventional therapies. Like any transformation process, initiation and development of PDCA are driven by a well known panel of genetic alterations, few of them are shared with most cancers, but many mutations are specific to PDAC and are partially responsible for the great inter-tumor heterogeneity. Importantly, this knowledge has been inefficient in predicting response to anticancer therapy, or in establishing diagnosis and prognosis. Hence, the pre-existing or rapidly acquired resistance of pancreatic cancer cells to therapeutic drugs rely on other parameters and features developed by the cells and/or the micro-environment, that are independent of their genetic profiles. This review sheds light on all major phenotypic, non genetic, alterations known to play important roles in PDAC cells resistance to treatments and therapeutic escape.

## 1 Introduction

**Fig. 1:**
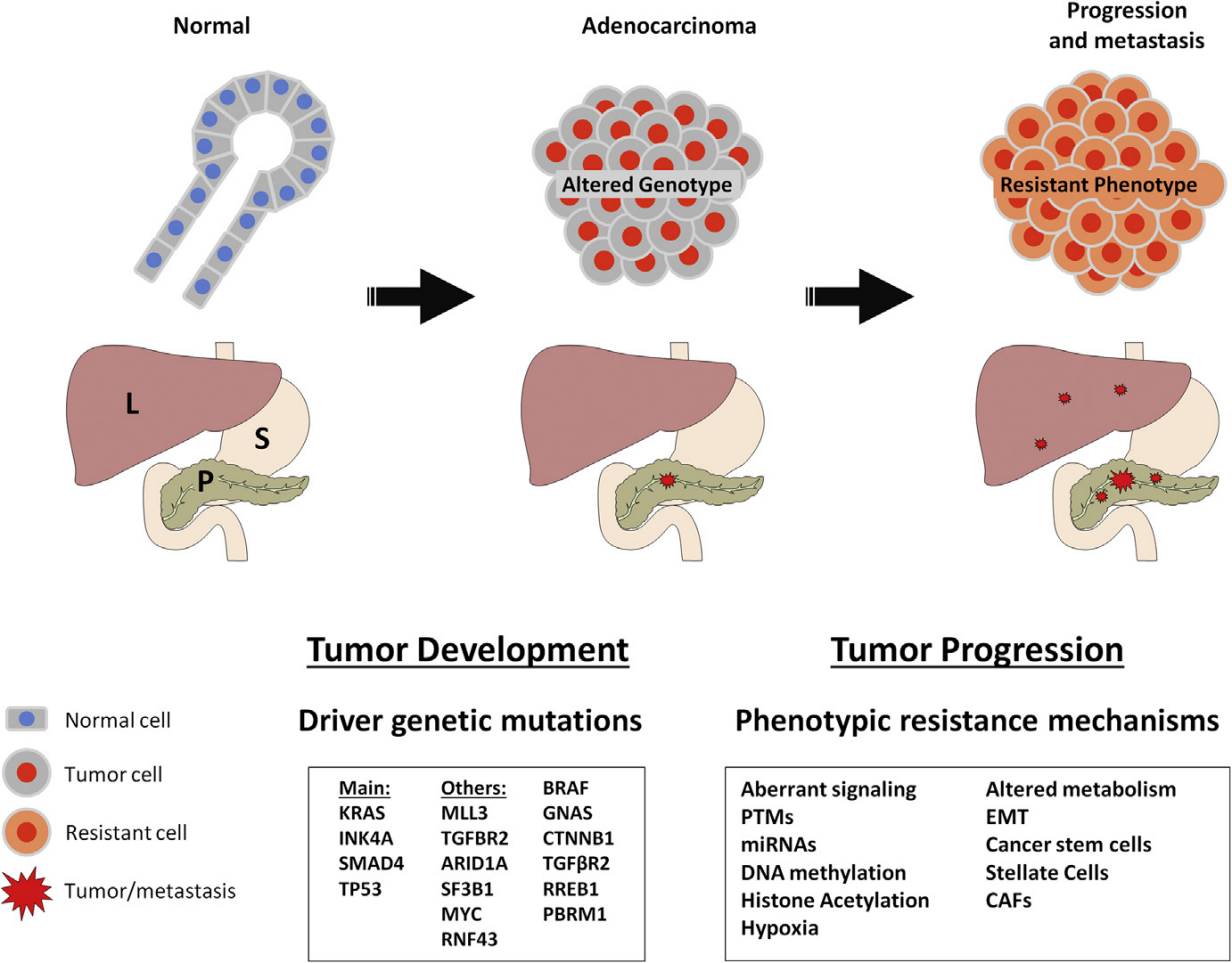
Schematic representation of the main genetic alterations driving the transformation of normal pancreatic tissue in to pancreatic adenocarcinoma, then a list of different phenotypic mechanisms controlling the process of chemoresistance, tumor progression and its metastasis to the liver. L: Liver; S: Stomach; P: Pancreas.

Pancreatic Ductal Adenocarcinoma (PDAC) is one of the most lethal human malignancies with a 5-year survival rate of 7% [1]. This extremely poor prognosis is due to the lack of active screening methods able to detect the tumor at early stages. Hence, the majority of patients are diagnosed at late metastatic or advanced stages, and only 10–15% of them are able to be resected surgically [2]. With or without surgery, patients are subjected to chemotherapy where gemcitabine was the baseline treatment for more than a decade and still employed nowadays [3]. Recently, combination of gemcitabine with nab-paclitaxel [4] and Folfirinox protocol [5] were able to improve the survival rates compared to the use of gemcitabine alone. Unfortunately, PDAC has a weak response to all current treatment regimens and many clinical trials targeting specific molecular pathways failed to improve this situation (Table 1). This fact is due to the development by the tumor of an extremely efficient cellular resistance to chemotherapeutic drugs. This resistance is supported by both cellular intrinsic and extrinsic factors related to tumor microenvironment [6]. Therefore, understanding these underlying mechanisms is an essential step to increase the efficacy of treatments and to decrease the mortality rate. Some studies focused on genetic mutations to explain the phenomenon of chemo-resistance in pancreatic cancer. However accumulating evidence leads us to look beyond the genetic alterations of the tumor and to consider the existing complex level of non genetic mechanisms driving resistance. This review aims to reveal the inaccuracy of relying only on genetic mutations to explain the extreme resistance of PDAC through focusing on all non genetic mechanisms including epigenetics, post translational modifications, aberrant signaling, altered metabolism, cancer stem cells, epithelial to mesenchymal transition and the cellular and non cellular components of the tumor microenvironment (illustrated in Fig. 1).

**Table 1:** List of all drugs targeting specific molecular pathways which, according to Clinicaltrials.gov, were tested at least one time in clinical trials for pancreatic cancer treatment and that gave results. None of them significantly improve the standard treatments.

Name	Alias	Molecular target	Pathway	Type
Aflibercept	L01XX44	VEGF	VEGF sign	rp
Alisertib	MLN-8237	AURKA	Cell cycle	i
Alvocidib	L86-8275	CDK9	Cell cycle	i
Apatorsen	OGX-427	HSP27	Proteostasis	as
Apricoxib	nd	COX-2	Multiple	i
Axitinib	AG-013736	VEGFR	VEGF sign	i
Bevacizumab	Avastin	VEGF	VEGF sign	ab
Bortezomib	PS-341	Proteasome	Proteostasis	i
Bosutinib	SKI-606	BCR-ABL + SRC family	Multiple	i
Bryostatin-1	nd	PKC	PKC	i
Cabozantinib	XL-184	HGFR + AXL + VEGFR	Multiple	i
Cetuximab	BMS 564717	EGFR	EGF sign	ab
Cixutumumab	IMC-A12	IGF-1R	PI3K/AKT	ab
Dactolisib	BEZ235	PI3K	mTOR	i
Dasatinib	BMS-354825	BCR-ABL, SRC family, c-KIT, EPHA2, PDGFRb	Multiple	i
Demcizumab	OMP-21M18	DLL4	Notch sign	ab
Erlotinib	L01XE03	EGFR	EGF sign	i
Etanercept	GP-2015	TNF α	TNF sign	ab
Everolimus	RAD001	mTORC1	mTOR	i
Galunisertib	(LY2157299)	TGF-beta-R1	TGF-beta sign	i
Ganetespib	(STA-9090)	HSP90	Proteostasis	i
Ganitumab	AMG 479	IGF1-1-R	PI3K/AKT	ab
Imatinib	STI571	BCR-ABL, c-KIT, PDGFR, DDR1/2, CSF1R	Multiple	i
Ipilimumab	MDX-010	CTLA-4	T-Lymphocyte activation	ab
Lapatinib	gw572016	EGFR	EGF sign	i
Motesanib	AMG 706	VEGFR	VEGF sign	i
nd	PF-00562271	FAK	Adhesion/invasion	i
nd	MK-2206	AKT1/2/3	PI3K/AKT	i
nd	RO4929097	Gamma-secretase	Notch sign	i
Olaparib	AZD-2281	PARP	DNA repair	i
Panitumumab	ABX-EGF	EGFR	EGF sign	ab
Panobinostat	nd	HDAC	Multiple	i
Pazopanib	GW-786034	VEGFR	VEGF sign	i
pertuzumab	R1273	HER2	EGF sign	ab
Pimasertib	AS-703026	MEK1/2	MAP kinase	i
Rabusertib	LY2603618	CHK1	Cell cycle	i
RAV12	RAAG12	Sodium channels (via N-linked carbohydrate antigen)	Cell homeostasis	ab
Romidepsin	FK-228	HDAC	Multiple	i
Ruxolitinib	INC424	JAK1/2	JAK-STAT	i
Saracatinib	AZD0530	SRC family	SRC	i
Selumetinib	AZD6244	MEK1/2	MAP kinase	i
Sirolimus	Rapamycin	mTORC1/2 (via FKBP12)	mTOR	i
Sorafenib	BAY-43-9006	VEGFR, PDGFR and Raf family kinases	Multiple	i
Sunitinib	SU-011248	RTKs	Multiple	i
Tanespimycin	17-AAG	HSP90	Proteostasis	i
Temsirolimus	CCI-779	mTOR	mTOR	i
Trametinib	GSK1120212	MEK1/2	MAP kinase	i
Vatalanib	PTK787	VEGFR, c-KIT	Multiple	i
Vismodegib	RG-3616	SMO (smoothened receptor)	Hedgehog sign	i
Vorinostat	MK-0683	HDAC	Multiple	i

ab: antibody; as: antisens; i: inhibitor, rp: recombinant protein.

## 2 Main text

### 2.1 Genetic mutations drive tumor progression but have a poor association with chemo-resistance

Genetic mutations in PDAC play a major role at the level of tumor initiation and development. PDAC arises from the stepwise progression of three types of precursor lesions called pancreatic intraepithelial neoplasm (PanIN), intraductal papillary mucinous neoplasm (IPMN), and mucinous cystic neoplasm (MCN) [7]. These precursor lesions are classified in to low-grade or high-grade [8]. The transformation of normal tissue to neoplastic lesions and then into invasive PDAC is orchestrated by the occurrence of successive key genetic mutations. *KRAS* gene encodes a RAS homolog belonging to the GTPase family. The amino acid substitution from glycine to aspartate in codon 12 of *KRAS* causes its constitutive activation observed in up to 95% of PDAC, representing the most common mutation in this cancer [9]. Oncogenic KRAS activates the MAP (Mitogen Activate Protein) kinase and the PI3 (Phosphoinositide 3) kinase pathways, leading to an increased proliferation. *KRAS* mutation is considered as an early event during pancreatic carcinogenesis occurring in 35% of low grade PanINs and reaching 75% in high grade PanINs [10]. This mutation facilitates the progression from low grade to high grade PanINs and is sufficient to initiate the PanIN lesions. *CDKN2A* (Cyclin Dependent Kinase Inhibitor 2A) is a tumor suppressor gene encoding the P16 protein that inhibits the phosphorylation of RB (Retinoblastoma Protein) protein and blocks cell cycle progression. The loss of this function leads to entry into mitosis and an increased cell division. *CDKN2A* is also frequently inactivated in 95% of PDAC patients [7]. It tends to arise at late stages of PanINs, with an incidence of 30–80% in low grade PanINs, and 92% in high grade PanINs. SMAD4 (SMAD Family Member 4), a main actor of TGF-beta signaling pathway, is mutated in 55 % of PDAC patients [11]. *SMAD4* expression is normal in low grade PanINs but it tends to be inactivated in late stages as it is the case in 31% of high grade PanINs [11]. *TP53* (Tumor Protein P53) tumor suppressor is inactivated in 75% of pancreatic cancers [7]. *TP53* mutation is a late event during PanIN progression to PDAC occurring in only 12% of high grade PanINs [12]. Additional detailed genetic analysis of PDAC led to the identification of new important genes which are mutated at lower percentage in these tumors. These genes include *RNF43* (Ring finger protein 43), *ARID1A* (AT-Rich Interaction Domain 1A), *TGFBR2* (Transforming Growth Factor Beta Receptor2), *GNAS* (GNAS Complex Locus), *RREB1* (Ras Responsive Element Binding Protein 1), *PBRM1* (Polybromo 1), *BRAF* (B-Raf Proto-Oncogene, Serine/Threonine Kinase), and *CTNNB1* (Beta Catenin 1) [13]. Some of these genes could be involved in tumor progression, but this has not been demonstrated yet. A subset of these mutations, some germinal but mostly somatic, affects genes involved in DNA damage repair (DDR) which provides susceptibility to develop cancer [14]. Importantly, deficiency in DDR was also shown to increase sensitivity to PARP (Poly (ADP-ribose) polymerase) inhibitors in different cancer types [15] and recent trials in PDAC patients tend to show a relative efficacy for some of them [16, 17].

Since mutations in these specific genes are considered to be the main drivers of tumor progression, scientists attempted to exploit them as therapeutic targets. However, after several trials, it has been evident that genetic mutations could be used as targets only in a small percentage of patients, firstly because the related drugs are not available or because the mutated genes can't be targeted by drugs. Some studies considered genetic mutations as critical component of drug resistance. A study by Fiorini et *al*. proposed that *TP53* mutations can drive the chemo-resistance to gemcitabine in pancreatic cancer cell lines [18]. However, a significant number of drug-resistant tumors were identified carrying no mutations in drug targets or activated pathways [19]. Several observations implied the participation of non-genetic mechanisms in drug resistance. A recent work performed in our laboratory [20] demonstrated that global genomic features (chromosomal instability index, mutation rate, copy number aberration, etc.) do not allow tumor classification or predict the response to therapy. Similar conclusions were obtained by Bailey's and coworkers who studied a larger cohort of patients using whole-genome sequencing approach [21]. In a recent study, drug sensitivities of 28 patient-derived cell lines and xenografts towards 305 chemical agents have been tested [22]. After combining different drugs, all individual tumors could be targeted efficiently. However, in one case only there was a correlation between the treatment efficacy and the genetic mutations, where the tumors containing a mutation in *STAG2* (Stromal Antigen 2) showed sensitivity to DNA cross-linking agents. Moreover, these tumors tend to be highly heterogeneous [23], composed of different cellular clones, each having different biological properties and different mutation profiles. This variety of clones often attenuates the response to treatment as the most resistant ones persist after chemotherapy and lead to relapse after a period of time [24]. Inter-tumor heterogeneity also plays an important role in response to drugs. This has been clearly demonstrated in a study where the sensitivity of five pancreatic cancer patient derived xenografts (PDXs) was tested towards MEK (Mitogen-activated protein kinase kinase) inhibitors. Despite the fact that all these PDXs carried the *KRAS* mutation they displayed different drug responses where two out of five PDXs were insensitive to MEK inhibitors [25]. For all these reasons, mutations solely are not reliable to explain the peculiar resistant phenotype observed in PDAC. Although a limited number of genetic mutations provoke tumor initiation and progression, the PDAC phenotype is highly heterogeneous. This suggests that this heterogeneity is engendered by genetic independent alterations, hence implying alterations at the epigenetic, transcriptomic, translational and post translational levels.

In accordance, transcriptome in PDAC was shown to be predictive of drug response and clinical outcome. A work combining laser capture micro-dissections followed by a detailed transcriptomic analysis revealed three main groups of patients named classical, quasi-mesenchymal and exocrine like, which were suggested to differ in terms of drugs sensitivities [26]. This has been confirmed by a complex computational approach on available transcriptomic data isolated from cancerous and normal gene expression profiles. This analysis led to the identification of two main tumor subtypes of which the basal like is having a poor clinical outcome [27]. Our group has studied PDAC samples from 17 patients that have been collected by endoscopic ultrasound-guided fine-needle aspiration or surgery and then xenografed in mice as PDXs and also kept as primary cultures of epithelial cells. Transcriptomic analysis has been performed using an Affymetrix approach and, without surprise, a significant heterogeneity in the mRNA expression profiles of tumors was observed. However, the bioinformatic analysis of these data was able to discriminate between patients with long-term and short-term survival which corresponded to patients with moderately or poorly differentiated PDAC tumors respectively [28]. Primary cultures of these tumors allowed the *in vitro* analysis of their sensitivity to the five most relevant anticancer drugs, thereby establishing individual profiles of drug sensitivity. Importantly, the response to the treatments was patient-dependent and the transcriptomic analysis revealed a drug-specific profile of sensitivity [28]. Similarly, a positive correlation was found between resistance to FK866, a specific inhibitor for NAMPT (Nicotinamide phosphoribosyl transferase), and the expression level of NAMPT mRNA [29]. Supervised clustering analysis of these transcriptomes identified a set of genes which are over expressed or under expressed in the resistant versus sensitive cells, confirming that the transcriptome is able to determine response to treatment. A supervised clustering analysis of the transcriptome of 55 patient derived xenografts indicated the presence of two clear subgroups defined as MYC-high and MYC-low depending on the expression level of MYC dependent genes. Results of tests performed in cell culture, spheroids, organoids and xenografts showed an increased sensitivity to JQ1, an inhibitor of bromodomain family of protein (to which MYC belongs to), in the panel of cells coming from the MYC-high subgroup [30].

The combination of these facts led us to concentrate on other more relevant molecular and non genetic mechanisms that better explain this drug resistance witnessed in PDAC.

### 2.2 PDAC intracellular mechanisms of chemo-resistance

#### 2.2.1 Altered metabolism

Altered metabolism contributes to the modulation of apoptosis, angiogenesis and drug targets thereby conferring a resistant phenotype [31]. Gemcitabine was and is one of the most used drugs for the treatment of PDAC patients. Evidence on the mechanisms of gemcitabine resistance has been accumulated during the last years. These mechanisms include the deficiency in ENT1 (Equilibrative Nucleoside Transporter 1) transporters, drug efflux by ABC (ATP-binding cassette) transporters, downregulation of the rate limiting enzyme deoxycytidine kinase (DCK), and the upregulation of RRM1/RRM2 (Ribonucleotide Reductase) that convert CDP (Cytidine diphosphate) to dCDP (Deoxycytidine diphosphate) [32]. Gemcitabine can also be inactivated either by deamination by cytidine deaminase or by dephosphorylation of the monophosphate form by 5′-nucleotidases [32]. Importantly, all these gemcitabine resistance mechanisms are independent of genetic alterations and are only due to altered expressions and/or activity of the involved enzymes. Other aberrations in classical metabolic pathways such as glycolysis, oxidative phosphorylation, and fatty acids metabolism have been shown to be also involved in chemo-resistance [31].

#### 2.2.2 Epigenetics

##### 2.2.2.1 DNA methylation and histone acetylation

Recently, the contribution of epigenetics in resistance mechanisms of pancreatic cancer gained more attention. DNA methylation plays a central role in regulating genes transcription, and its deregulation is associated with tumorigenesis. A study by Ramachandran et *al*. reported that the silencing of *PYCARD* (PYD And CARD Domain Containing) gene, which encodes TMS1 (Target of methylation induced silencing 1), through the methylation of its promoter, is involved in resistance of pancreatic cancer [33]. Inversely, the over-expression of TMS1 via recombinant gene expression in PDAC cells or, treating these cells with the methylation inhibitor 5-azacytidine, increased their sensitivity to gemcitabine and docetaxel [33]. Histone acetylation may also contribute to pancreatic cancer cells resistance. This has been shown by Ono et *al*. who demonstrated that the HAT (Histone acetylase) p300 confers resistance to gemcitabine whereas its inhibition by C646 (HAT inhibitor) decreased histone acetylation and improved the sensitivity to gemcitabine [34].

##### 2.2.2.2 MiRNAs and lncRNA

MiRNAs (micro RNAs) are double stranded RNAs of 18–24 nucleotides. They bind mRNAs (messenger RNA) to regulate their expression. Their abnormal production may contribute to resistance in cancer cells. Moriyama et *al*. [35] found that miR-21 is upregulated in pancreatic cancer cells and its inhibition decreased their proliferation, invasion and chemo-resistance. A comparison of miRNA profiles of gemcitabine resistant and gemcitabine sensitive pancreatic cancer cells identified 33 differentially regulated miRNAs. One of them was miR-497, which was downregulated in resistant cells, and its upregulation enhanced the sensitivity of PDAC cells to gemcitabine and erlotinib [36]. miR-33a was found to be downregulated in gemcitabine resistant cells [36] and its overexpression was able to sensitize pancreatic cancer cells to gemcitabine and inhibit tumor growth by suppressing the expression of Pim-3 kinase [37]. MiR-181b enhances the sensitivity of pancreatic cells to gemcitabine both in vitro and in vivo by downregulating BCL2 [38]. MiR-506 downregulation helps in avoiding apoptosis and induces chemo-resistance in pancreatic cancer cells through SPHK1/Akt/NF-Κb signaling pathway [39]. Both miR-211 and let-7 enhance the sensitivity to gemcitabine by downregulating RRM2 (altered metabolisms section), which is an important target of gemcitabine in pancreatic cancer cells [40].

lncRNAs are long non coding RNA recognized as important epigenetic regulators by acting as sponges for miRNAs [41]. Some lncRNAs may be involved in PDAC such as HOTTIP (Homebox A transcript at the distal tip) which has been found upregulated in PDAC by a lncRNA microarray profiling and whose knockdown was able to sensitize PDAC cells to gemcitabine treatment [42]. Similar findings have been described regarding the lncRNA ROR (Regulator of reprogramming) whose knockdown by siRNA (Small interfering RNA) strategy could sensitize two PDAC cell lines to gemcitabine whereas its overexpression conferred resistance to gemcitabine [43].

#### 2.2.3 Post translational modifications

When PDAC cells are subjected to chemotherapy, stress response pathways are activated. These responses are tightly regulated by specific post-translational modifications (PTMs) of involved proteins and, alterations of this system resulting in the over-activation of these stress responses can contribute substantially to resistance [44]. Looking for such alterations of PTMs in response to gemcitabine, we found that the pro-survival role of SNIP1 (Smad nuclear interacting protein 1) was enhanced upon gemcitabine treatment by a mechanism involving the activation of P38 MAP kinase which phosphorylates SNIP1 at Ser35. This phosphorylation allowed the sumoylation of SNIP1 at Lys30 which favored cell survival [45].

#### 2.2.4 Signaling pathways

Aberrant signaling pathways are a main hallmark of tumorigenesis and drug resistance. Wnt pathway is often over-activated in PDAC and this plays an important role in drug resistance. WNT5 expression causes gemcitabine resistance in a xenograft model of PDAC [46]. Masitinib, a tyrosine kinase inhibitor, can sensitize PDAC cells to gemcitabine through the inhibition of Wnt/β-Catenin pathway [47]. Accumulating evidences also demonstrated the role of Notch pathway in chemo-resistance. NOTCH2 (Neurogenic Locus Notch Homolog Protein 2) and JAG1 (Jagged1) are upregulated in gemcitabine resistance PDAC cell lines [48], and the knock-down of NOTCH1 expression by siRNA enhanced the sensitivity of PDAC cells to gemcitabine [49]. Hedgehog pathway is also over-activated in PDAC and results in formation of a dense desmoplasia. Studies using KPC mouse model of PDAC identified the important role played by the Hedgehog pathway in resistance as the desmoplastic reaction resulted in a decreased delivery of systemic gemcitabine into the tumor [50]. NFκB (Nuclear Factor Kappa B Subunit) is another pathway constitutively activated in PDAC. The downregulation NFκB and its downstream targets such as survivin, BCL-XL (B-cell lymphoma-extra large), XIAP (X-linked inhibitor of apoptosis protein) and CIAP (Cellular inhibitor of apoptosis protein), by 3,3-diindolylmethane (DIM) was able to potentiated the efficacy of different anti-cancer drugs *in vivo* and *in vitro* [51, 52]. The MAP kinase signaling pathway may also contribute to the chemo-resistance to certain treatments [53]. Indeed, resistant cell lines shows higher ERK activity than sensitive cells and the inhibition of this pathway resulted in an increased 5-FU sensitivity, but an increased resistance to gemcitabine [53]. Finally, PI3K/AKT has been shown to be also one of the mediators of chemo-resistance to gemcitabine in pancreatic cancer, and the use of PI3K inhibitors like wortmannin and LY294002 increased the sensitivity of PDAC cells to gemcitabine [54].

### 2.3 Resistance's mechanisms ruled by the tumor microenvironment

Tumor microenvironment is the interstitial tissue that surrounds cancer cells and is composed of different cell types including pancreatic stellate cells, cancer associated fibroblasts, nerve cells, endothelial cells, and inflammatory cells, in addition to extracellular matrix components. These cellular and non cellular components can confer drug resistance through diverse actions [55].

#### 2.3.1 Extracellular matrix (ECM)

PDAC is characterized by the overproduction of ECM components leading to fibrosis, which is mainly mediated by TGFβ signaling pathway [56]. These components interact with cancer cells resulting in the formation of a dense desmoplasia that may favor cell migration, invasion, and also chemo-resistance by preventing proper drug entrance in the tumor thereby limiting the delivery to tumor cell. Several studies demonstrated the important roles of key extracellular components like Collagens (I, III, IV), Hyaluronan, Decorin, Versican, Fibronectin, Laminin, and Osteonectin/SPARC in the process of chemo-resistance [57]. Hyaluronic acid (HA) is a glycosaminoglycan (GAG) and is a major component of the normal ECM. It was found to be increased in several tumor types, including PDAC [58], and was shown to be significantly involved in tumor progression, chemo-resistance, and poor prognosis [59]. Based on these findings, a pegylated recombinant human hyaluronidase (PEGPH20), the enzyme that breaks down HA in stroma, has been developed and demonstrated highly promising preclinical and clinical anti-tumor activity in PDAC [60].

#### 2.3.2 Cancer stem cells (CSCs)

Like other tumor types, PDAC tumors contain cancer stem cells which are able to self renew and to give rise to new different cell populations that form the tumor mass [61]. These pancreatic CSCs have been reported to be responsible not only for tumor recurrence but also for metastatic spread and, importantly, for chemo-resistance of the tumor [61]. Importantly, establishment of gemcitabine-resistant pancreatic cancer cell lines showed that resistant cells were enriched in cells with CSCs markers [62].

#### 2.3.3 Pancreatic stellate cells (PSCs)

Accumulation of reactive oxygen species (ROS), cytokines, and growth factors secreted by the tumor cells activate PSCs stimulating them to differentiate into myofibroblast phenotype secreting extracellular matrix components [63]. Factors secreted by pancreatic stellate cells produce a chemo-resistant phenotype and increase the survival of PDAC cells by preventing the H_2_O_2_-induced apoptosis and by decreasing the sensitivity of tumor cells to both gemcitabine and radiations [64]. One of these factors has been identified as Periostin which is able to increase gemcitabine resistance [65]. Pancreatic cancer cells cultured with ECM proteins produced by PSCs display an increased resistance to 5-FU, cisplatin and doxorubicin [57]. A recent study confirmed the role of PSCs in radio-resistance by activating the integrin-FAK (Focal adhesion kinase) signaling in tumor cells [66].

#### 2.3.4 Cancer associated fibroblasts (CAFs)

CAFs represent the majority of the cellular compartment of tumor microenvironment of PDACs. A study by Richard's et al. showed that CAFs exposed to chemotherapy play an active role in the survival and proliferation of cancer cells. They also found that CAFs are intrinsically resistant to gemcitabine. Furthermore, exposition of CAFs to gemcitabine significantly increased the release of exosomes, a type of extracellular vesicles. These exosomes tended to increase the expression of SNAIL in recipient epithelial cells, thereby promoting proliferation and drug resistance. Importantly, treatment of gemcitabine-exposed CAFs with an inhibitor of exosome release, called GW4869, significantly reduced the survival in co-cultured epithelial cells, pointing at an important role of CAFs' exosomes in chemo-resistance [67]. Part of the mechanism by which exosomes confer chemo-resistance in pancreatic cancer cells has been revealed and involves the upregulation of two ROS detoxifying enzymes, the superoxide dismutase 2 (SOD2) and catalase (CAT), and the miR-155-mediated downregulation of gemcitabine-metabolizing enzyme, DCK [68].

#### 2.3.5 Immune cells and inflammation

Immune cells, such as tumor associated macrophages (TAMs), can affect the response of tumor cells to chemotherapy through a process called environment-mediated drug resistance. A study by Amit et al. showed that TAMs can secrete the enzyme cytidine deaminase which metabolizes gemcitabine into its inactive form (2′,2′-difluorodeoxyuridine) thereby leading to the survival of cancerous cells and favoring the emergence of chemo-resistant clones [69]. Cytidine deaminase may have unexpected origin as it has recently been shown that intra-tumor bacteria, mainly belonging to Gammaproteobacteria, also express and secrete a bacterial form of this enzyme which is active on gemcitabine [70]. Inflammation within the pancreatic tumor environment has been linked to chemo-resistance and tumor progression through NFκB, IL6 (interleukin 6), Toll like receptor and TGFβ pathways [71].

#### 2.3.6 Hypoxia

PDAC is a compact solid tumor with reduced blood flow that leads to temporary or chronic hypoxia [72]. Such Hypoxic conditions stabilize HIF1A (Hypoxia inducible factor 1 alpha) which is known to participate in the resistance to chemotherapy and radiotherapy [73]. Moreover, most chemotherapies induce their toxicity also through the generation of ROS. As the generation of ROS is strongly reduced in cells under hypoxia the efficacy such treatments are also reduced [74, 75]. Hypoxia can increase the expression of P-glycoprotein, the product of multidrug resistance gene (MDR1), which is involved in drug inactivation and consequently in drug resistance [76].

#### 2.3.7 Epithelial to mesenchymal transitions (EMT)

EMT is a process in which epithelial tumor cells lose their epithelial markers like E-cadherin and start expressing mesenchymal markers like vimentin, undergo cytoskeletal remodeling followed by loss of cell polarity and acquisition of an invasive phenotype which aids the metastatic process [77]. Recent studies suggest an important role of EMT in resistance to gemcitabine, 5-FU, and cisplatin, which can be reversed by silencing of ZEB1 (Zinc finger E-box-binding homeobox 1) in resistant PDAC cancer cell lines [78, 79].

## 3 Conclusion

Despite the fact that genetic mutations are responsible for the tumor development, they cannot explain the phenomenon of resistance nor help to anticipate the response to a given chemotherapeutic drug. PDAC has a well known set of mutations but, nevertheless, it displays an incredible inter-tumor and intra-tumor heterogeneity. This may explain why the past attempts to target mutations to surpass resistance and to provide more efficient cures for PDAC didn't reach satisfactory results. Hence, it becomes obvious that other paths must be taken in order to solve the mystery of PDAC resistance and to design better treatments able to improve the survival rates. The literature contains an increasing number of examples showing that resistant mechanisms are associated with a particular phenotype of the tumor, both at the tumoral cell level and at the associated microenvironment level (Fig. 1). So, deeper investigations of these resistance mechanisms will reveal new molecular pathways that could be targeted either by already available molecules or by new specifically designed ones to ultimately improve the survival of PDAC patients.

## Declarations

### Author contribution statement

All authors listed have significantly contributed to the development and the writing of this article.

### Funding statement

This work was supported by La Ligue Contre le Cancer, INCa, Canceropole PACA, DGOS (labellisation SIRIC) and INSERM. Mirna Swayden was supported by La Ligue Contre le Cancer and the Lebanese Ministry of the Interior and Municipalities.

### Competing interest statement

The authors declare no conflict of interest.

### Additional information

No additional information is available for this paper.
